# Healthy and Sustainable Food: A Cross-Cultural Study among Spanish and Italian Consumers

**DOI:** 10.3390/foods11131941

**Published:** 2022-06-29

**Authors:** Debora Scarpato, Giacomo Rotondo, Mariarosaria Simeone

**Affiliations:** 1 Department of Economics and Legal Studies, University of Naples Parthenope, 80133 Napoli, NA, Italy; debora.scarpato@uniparthenope.it (D.S.); giacomo_rotondo@yahoo.it (G.R.); 2 Department of Law, Economics, Management and Quantitative Methods (DEMM), University of Sannio, Via delle Puglie 82, 82100 Benevento, BN, Italy

**Keywords:** consumer behavior, food safety, environmental attributes, food choices, organic products, certifications

## Abstract

The purpose of this paper is to analyse the perception of environmental attributes and food safety in Italy and Spain and how these different perceptions influence the importance of various attributes and certifications related to food. These two countries have a common root and some undeniable similarities, but there are two completely distinct individualities. Three main research questions have, thus, been identified: attention to food safety regarding Italian and Spanish consumers; the role of ecological certifications in the perception of health in the two countries; the role of the brand and the choice of organic products in the perception of food safety. To answer the research questions, a questionnaire was administered, face to face, to a sample of both Spanish consumers and Italian consumers. The analysis of the data collected through the questionnaires was administered using two logistic regressions to identify which variables influence issues concerning the health and safety of food products. A Spanish consumer interested in ecological certifications is also a consumer who is attentive to the safety and health of food consumption. Looking at Italian consumers, it is highly probable that a consumer who assesses ecological certifications positively is also a consumer concerned about food safety, but this probability increases the likelihood this consumer’s choices are guided by brands and frequent consumption of organic products. This research has allowed us to highlight sharp differences in the approach to consumption in these two countries, which, despite similar cultures and the same sensitivity to environmental and food safety issues, exhibit marked differences in the selection of food products.

## 1. Introduction

Italy and Spain have a common root and some undeniable similarities, but concretely they represent two completely distinctive individualities. The aim of our research was to understand how the perception of food safety and environmental attributes is different in these two countries and how this different awareness influences the importance of attributes and certifications related to food.

There is an increasingly significant need to aim for the adoption of sustainable consumption models that are capable of ensuring good nutrition with a low environmental impact [1,2,3]. This objective is multi-dimensional, because it encompasses nutritional, environmental and socio-economic aspects [4].

In terms of nutritional aspects, food security can be studied by focusing on two main dimensions. The first is procurement, also known as Food Security, which concerns continuous access to a diet that is sufficient in terms of quality, quantity and variety, for a healthy and active life [5,6,7]. Food security is usually measured as a combination of several indicators, among which food availability and food access are particularly important [8]. In terms of the dimension of food insecurity, food safety can be understood as the inverse of food risk, in that food safety becomes the probability of not suffering some hazards from eating the food in question [9]. In the broadest sense, “insecurity” is derived from the combined effect of food attributes capable of influencing consumer health at the time of consumption or during subsequent stages [8]. In other words, these definitions include aspects related to food composition (toxicology), informational aspects (i.e., information provided to consumers about food characteristics or methods/quantity of consumption) and nutritional aspects. In addition, there is a broader perspective that frames food safety in relation to quality of life and the environment, on which the negative externalities of industrial agriculture and intensive breeding have an impact [10]. In the same way, all modern food transformation and distribution activities in global supply chains are potential negative externalities to be considered.

Food systems are simultaneously a leading cause of environmental degradation and depletion of natural resources. Food systems are responsible for a significant share (20–33%) of greenhouse gas (GHG) emissions, and are a major driver of land conversion, deforestation and loss of biodiversity [11]. Agriculture alone accounts for roughly 70% of global freshwater withdrawals, and causes water pollution. With the world’s population predicted to expand to 9.7 billion individuals by 2050, these environmental pressures and impacts do not make current food systems sustainable. In 2014, the FAO/WHO Second International Conference on Nutrition (ICN2) recognized that: “*current food systems are being increasingly challenged to provide adequate, safe, diversified and nutrient rich food for all that contribute to healthy diets due to, inter alia, constraints posed by resource scarcity and environmental degradation, as well as by unsustainable production and consumption patterns, food losses and waste, and unbalanced distribution*”. So, shaping food systems for Sustainable Healthy Diets also requires consideration of the environment. In particular, “*Sustainable Healthy diets are dietary patterns that promote all dimensions of individuals’ health and wellbeing; have low environmental pressure and impact; are accessible, affordable, safe and equitable and are culturally acceptable*” [12,13].

Many consumers are not fully aware of the association between food consumption and environmental consequences. Some food companies are developing labels to inform consumers of the environmental impact of production. Good product labels can provide consumers with rich, specific, expert-certified product information, and expert labels might play an important role as trusted sources of information in an increasingly complex global food system [14].

In Spain, a study conducted on 17 autonomous regions suggests the presence of relatively good dietary habits [4]. In particular, the results of one study, the main objective of which was to understand the perception and attitude of the Spanish consumer towards environmental labels and their impact on purchasing decisions, has shown that even while consumers have a positive attitude towards the attributes of sustainability in food products, there are constraints as to how this is manifested in their purchasing behaviour [15].

Another interesting study in Spain has shown that consumers are increasingly concerned about the environmental and food safety issues of food products, and that the demand for organic products is growing as a result. The findings indicate that consumers who are concerned about healthy diets and environmental degradation are the most likely buyers of organic products and are willing to pay higher prices [16].

The rapid growth in the market for organic products can probably be attributed to the general perception among customers that these foods are healthier and more nutritious than conventional foods [17,18,19]. According to Aschemann Witzel, Jessica, and Stephan Zielke [20], the price is the major perceived barrier to purchase, and this is why support for organic farming is still a good policy for improving sustainability.

This is happening in a similar manner with respect to the consumption of lamb meat in Spain. Indeed, in this scenario, the greater potential for growth is witnessed in the context of the consumption of organic lamb meat. A study conducted by Rabadán et al. [21] shows that the main reasons for non-consumption of lamb meat relate to supply, higher price and lack of any guarantees that the meat is organic. In other words, increasing the consumption of organic lamb meat necessarily requires greater effort in the supply chain and raising more awareness about the controls that guarantee certified organic origin.

Sustainability has become one of the most important challenges for wine companies over the last ten years. From a marketing point of view, sustainability can be seen as a way to differentiate wines in order to meet the specific needs of each market segment and may even be considered necessary to ensure the future growth of this sector. The results of a study conducted by Sellers [22] in order to determine the price consumers are willing to pay for a sustainable wine, as compared to a conventional wine with similar characteristics, show that there is a premium price Spanish consumers are willing to pay for a sustainable wine, and that there are differences within the main market segments.

The use of chemical substances in agriculture entails risks for both human health and the environment. Voluntary and mandatory regulatory measures have been introduced to promote a reduction in pesticide use. The proliferation of these types of standards is related to consumers and their increasing interest in foods with a low negative impact on health and the environment. The study of Stranieri et al. [23] investigates the interaction between healthy, ecological, ready-to-use foods and the factors determining the purchase of food products with ecologically sustainable attributes. According to their findings, consumers show great concern for agricultural practices, and the most concerned consumers would prefer much more stringent environmental regulations.

With regards to Italy, on the other hand, we should first note how the established culinary tradition is rooted in regional cuisine. These strong traditions and the excellence of local production have probably slowed the trend towards globalization of consumption models in Italy [23,24,25]. Nevertheless, Italy’s food consumption models seem to be evolving to reflect the significant changes in Italian lifestyles and dietary habits witnessed in recent decades. Among these, we can cite the increase in meals away from home, the widespread presence of take-away and roadside food, not to mention the growth in a recreational attitude towards food, especially among young people [26,27]. We are witnessing a food polytheism, to which must be added the rising demand in terms of food variety, food security and attention to environmental sustainability. In recent years, there has been a greater consumer orientation towards health and the environment [28,29]. People are more attentive to the foods they eat. They often prefer healthy foods to those that could be harmful to their health, and they are also very attentive to the environmental sustainability of foods and food packaging.

In Italy, an annual study carried out by LifeGate together with Eumetra MR (2019) showed that 36 million people were sensitive to this issue (almost twice as many as in 2015, when 21 million people were “interested”), half of which were environmentalists. This net increase of interest is determined by climate change and the much more frequent occurrence of natural catastrophes than in the past, as well as the increasing involvement of generation Z, which, fearing for their future and that of the planet, are very assertive in fighting for more sustainable behaviour by the entire world population. However, Generation Z are not the only ones interested in this theme. Women and college graduates have also exhibited great interest in this topic.

To this purpose, three main research questions have been thus defined: the role of ecological certifications in the perception of health in the two countries of Spain and Italy; attention to food safety among Italian and Spanish consumers; the role of brand and the choice of organic products in the perception of food safety.

A questionnaire was administered, face to face, to a sample of Spanish consumers in the area of Segovia and to a sample of Italian consumers in Benevento.

The analysis of the data collected through the questionnaires was administered in Italy and Spain using two logistic regressions to identify which variables influence concern about the issues of health and safety of food products. The results that arose from this empirical analysis were interesting.

This article is divided into several sections. Following this analysis of the reference literature, which forms the foundation for this study, we have the Material and Methods section, which explains the data collection method, which was a questionnaire administered to a reasonable sample of consumers. The data were then analysed through logistic regression, as reported in the Results section of the empirical analysis. The study concludes with the Conclusion and Discussion section, which presents the main conclusions that emerged from this analysis of two samples of consumers and the policy implications which arise from this research.

## 2. Material and Methods

A questionnaire was administered to a sample of Spanish consumers from the city of Segovia and to a sample of Italian consumers from the city of Benevento.

The questionnaire was divided into 4 sections and the selection of variables came from the analysis of many studies. In the first section, we investigated the importance of food safety, appearance and origin and some of the variables collected from the study of Kaczorowska et al. [30]. In the second section of the questionnaire, we proceeded to examine the role of brands in the choice of products, while, in the third section, we investigated attention to social responsibility, ecological certifications and knowledge of organic products. In particular, these variables were selected looking at the study of Haas et al. [31], published in the Journal Foods (2021), which highlighted differences in the rating of food safety and quality cues between consumers from Kosovo (Prishtina) and consumers from Albania. Finally, in section 4 of the questionnaire, we considered consumers’ purchasing behaviour with respect to biological, fair trade and organic products [32].

Regarding the ethical aspects, all subjects gave their informed consent for inclusion before they participated in the study and data were treated anonymously. This study was not a medical study and, since it did not involve sensitive data (political, religious, medical data), we could collect data by simply informing participants about the goal of the research and the use of data.

Table 1 shows the ten variables studied in the two samples.

The interviews took place *face to face* and were administered to two sample populations representing Spain and Italy in June of 2021. In total, 366 questionnaires were administered. In particular, interviews were conducted in Benevento (Italy) and in Segovia (Spain) The Spanish sample consisted of 175 consumers, 45% women and 55% men, who were all university students. The Italian sample was represented by 191 consumers, with the same gender-based composition, in terms of the consumers interviewed, as in the Spanish sample. Both the Italian and the Spanish samples were aged between 20 and 25 years old.

These findings can be considered as exploratory research conducted in area[s] with similar characteristics, in Italy and in Spain, but without inferential objectives. Data were analysed using two logistics regressions. 

Binary logistic regression was used to analyse the other variables being studied in the two samples.

The fundamental form of a binary logistic regression equation with K independent variables is [33,34]:

$$\hat{L}=\alpha +\beta_{1}X_{1}+\beta_{2}X_{2}+\ldots +\beta_{K}X_{K},$$

with $\hat{L}=\ln\left[\frac{P\left(Y=1\right)}{1-P\left(Y=1\right)}\right]$ indicating the logit.

This equation indicates that the expected value of the natural logarithm of the ratio between the two probabilities associated with the dichotomy is a linear function of the selected independent variable *K*, which can, in turn, assume a value of either 1 or 0:

If we take the anti-logarithm of this equation, we have:

$$e^{\hat{L}}==\frac{P\left(Y=1\right)}{1-P\left(Y=1\right)}e^{\alpha +\beta_{1}X_{1}+\beta_{2}X_{2}+\ldots +\beta_{K}X_{K}},$$

i.e., a formulation that allows the effect of each independent variable on the odds of *P*(*Y* = 1) to be isolated from its reciprocal [1 − *P*(*Y* = 1)]. For independent dichotomous variables (with values 1 and 0), the exponential of regression coefficients β indicates, for each independent variable, the odds ratio when it transitions from value 0 to value 1, while the remaining independent variables assume a value of 0. For variable *X*_1_, for example, we have:

$$\frac{{\left[\frac{P\left(Y=1\right)}{1-P\left(Y=1\right)}\right]}_{X_{1}=1}}{{\left[\frac{P\left(Y=1\right)}{1-P\left(Y=1\right)}\right]}_{X_{1}=0}}=\frac{e^{\alpha +\beta_{1}\left(1\right)}}{e^{\alpha +\beta_{1}\left(0\right)}}=e^{\beta_{1}\left(1\right)-\beta_{1}\left(0\right)}=e^{\beta_{1}}.$$

Starting from

$$\left[\frac{P\left(Y=1\right)}{1-P\left(Y=1\right)}\right]=e^{\alpha +\beta_{1}X_{1}+\beta_{2}X_{2}+\ldots +\beta_{K}X_{K}},$$

the expected probability *P*(*Y* = 1) can be isolated:

$$P\left(Y=1\right)=e^{Z}\left[1-P\left(Y=1\right)\right],\;\mathrm{with}\;Z=\alpha +\beta_{1}X_{1}+\beta_{2}X_{2}+\ldots +\beta_{K}X_{K},$$

from which is obtained

$$P\left(Y=1\right)+e^{Z}\left[P\left(Y=1\right)\right]=e^{Z}$$

$$\left(1+e^{Z}\right)P\left(Y=1\right)=e^{Z}$$

$$P\left(Y=1\right)=\frac{e^{Z}}{1+e^{Z}}$$

This last formulation allows us to verify the effect that the variation of each independent variable—in the case of dichotomous variables, as in our analysis, from the transition of these from mode 0 to mode 1—exerts on the dependent variable, i.e., on the probability of a given event occurring, *P*(*Y* = 1). In our study, *P*(*Y* = 1) indicates the probability that the consumer is attentive to the quality and healthiness of food products.

The expected probability *P*(*Y* = 0) = 1 − *P*(*Y* = 1) is seen to be equal to:

$$P\left(Y=0\right)=\frac{\frac{e^{Z}}{1+e^{Z}}}{e^{Z}}=\frac{1}{1+e^{Z}}$$

The methodological description just provided makes reference to dimensions such as odds and odds ratio. These are defined as follows. The odds are defined as the ratio between two probabilities, i.e., between the probability of the event occurring and the probability of non-occurrence. The odds ratio is the simple ratio between the two odds.

The section analysing the empirical findings includes sample-related numerical data referring to both—the odds and the odds ratio. It is important to clarify these values because, as we shall see, the results obtained through logistic regression may be interpreted in reference to the odds ratios.

The estimation of the logistic regression parameters, *β* and *α*, uses Maximum Likelihood Estimation.

## 3. Results of the Empirical Analysis

After selecting food safety as the dependent variable, the nine independent variables were described and encoded. This gave us ten binary variables.

The Spanish sample consisted of 45% women and 55% men and 75% of the sample of Spanish were worried about food safety.

The distribution of cases regarding personal concerns with food safety issues among Italian consumers showed that 79.1% of consumers interviewed were worried about food safety.

A comparison of the two samples showed how food safety worries Italian consumers slightly more than Spanish consumers: 79 out of 100 consumers for the Italian sample, versus 75 out of 100 consumers for the Spanish sample.

### 3.1. Spanish Sample Analysis

The application of binary logistic regression to the Spanish sample produced the results summarized in Table 2

As for the interpretation of the results [35], the values reported in the Exp column (B) provide the odds ratio for exhibiting particular attention to food health and safety:

- the first 5 independent variables express the importance of product attributes in consumer choices;
- the organic certification variable expresses the importance of organic certifications in consumer choices;
- the fair trade, local food, organic products variables express the importance of these factors when products are purchased.

Proceeding with the evaluation of the odds that a consumer attentive to pricing in consumer choices is interested in the health and safety of food products, the odds were 0.747 times that such a consumer attributed no importance to pricing.

For the variables relating to the characteristics of food products, we found that:

- a consumer who considered product appearance to be important had 1.364 higher odds of being attentive to food safety than a consumer who, instead, did not allow product appearance to affect them;
- a consumer who gave importance to the “origin” attribute had only 1.019 higher odds of being interested in food health than a consumer who did not consider origin to be an important attribute;
- a consumer who cared about the brand had 0.634 lower odds of experiencing a particular concern about food safety than a consumer who was not brand attentive;
- Finally, a consumer who assessed the presence of ecological certifications positively had 2.931 higher odds of being worried about issues related to product safety and healthiness than a consumer who was not interested in certifications.
- The odds of being attentive to Food Safety were greater for a consumer who ignored the meaning of organic certification, relative to a consumer who understood its meaning and usefulness.

Finally, the odds of being worried about issues related to the healthiness of food products were increasingly higher for consumers who often bought organic, fair trade and local products, as compared to consumers who claimed not to consume such products.

Observing the values in the table, we notice how the variable for which value B is significant identifies Environmentally Friendly (Sig. = 0.007), while for the other variables, B is not significantly different from zero, and there is a high probability of making a type I error.

Implementing logistic regression once again, therefore, by reporting Environmentally Friendly as the only independent variable, we obtained the results summarized in Table 3, as shown below.

As seen in Table 3, the odds of being a food safety consumer were 3.27 times higher for consumers who considered ecological product certification to be important, as compared to consumers who were not interested. The value of B, in relation to the Environmentally Friendly variable, can be used to determine the expected probability of a consumer being attentive to food health and safety. Furthermore, the likelihood of a consumer who was concerned about the environmental impact of the products they consumed also exhibiting a particular sensitivity to food safety issues was equal to (The initials EF, which appear in the subscript of the calculated probability estimate, indicates the variable relating to the presence of environmental certifications.):

$$\mathrm{Pr}ob_{EF}\left(y=1\right)=\frac{e^{0.480(1)+1.187(1)}}{1+e^{0.480(1)+1.187(1)}}=0.83.$$

In conclusion, in 83 out of 100 cases, a Spanish consumer interested in ecological certifications was also a consumer who was attentive to the safety and health of food consumption.

### 3.2. Italian Sample Analysis

In reference to the sample of **Italian consumers**, logistic regression, through the application of Forward Stepwise (Likelihood Ratio) [36], made it possible to identify four independent variables which, exhibiting a significantly non-zero coefficient B (Table 4), affected the estimated probability of a consumer exhibiting particular attention to Food Safety topics.

Before calculating the estimated probabilities, the Exp(B) values showed how:

- for the case of a consumer interested in ecological certifications, the odds of being a food safety-conscious consumer were a full 1697 times higher than the odds for a consumer who was not interested in ecological certifications;
- the odds of being worried about product health and safety were, for the case of a brand-attentive consumer, 59 times higher than the odds for a consumer not interested in the brand;
- the odds of being attentive to food safety for a consumer who consumed organic products were 4.9 times the odds for a consumer who did not consume organic products;
- finally, the variable aspect of the products: the odds of a consumer being attentive to product safety and health were, for a case of a consumer who took product appearance into consideration, lower than the odds for a consumer who gave no importance to product appearance.

It is possible to calculate, in relation to the four independent variables that were selected, the following estimated probabilities of being a consumer attentive to food product health and safety (The initials appearing in the subscript of the calculated probabilities, which refer to product characteristics that can influence both their purchase and consumption behavior, are the following respective indicators: *EF*, in the presence of environmental certifications; *A*, appearance; *B*, brand; *OP*, consumption (frequent) of organic products.).

The probability that a consumer interested in ecological certifications was also concerned with food safety was:

$$\mathrm{Pr}ob_{EF}(Y=1)=\frac{e^{-5.418(1)+7.437(1)}}{1+e^{-5.418(1)+7.437(1)}}=0.8827$$

The probability that a consumer who let the brand influence them was also attentive to food safety was:

$$\mathrm{Pr}ob_{B}(Y=1)=\frac{e^{-5.418(1)+4.075(1)}}{1+e^{-5.418(1)+4.075(1)}}=0.207$$

The probability that a consumer who often consumed organic products was attentive to food safety was:

$$\mathrm{Pr}ob_{OP}(Y=1)=\frac{e^{-5.418(1)+1.587(1)}}{1+e^{-5.418(1)+1.587(1)}}=0.021$$

The probability that a consumer who gave importance to product appearance paid attention to food safety issues was:

$$\mathrm{Pr}ob_{A}(Y=1)=\frac{e^{-5.418(1)-2.290(1)}}{1+e^{-5.418(1)-2.290(1)}}=0.000449$$

It was highly probable that a consumer who assessed ecological certifications positively was also a consumer concerned about food safety. This probability increased when, in addition to appreciating the presence of ecological certifications, a consumer claimed that their consumer choices were guided by brands and he or she consumed organic products frequently, as shown below:

$$\mathrm{Pr}ob_{EF,B}\left(y=1)\right)=\frac{e^{-5.418(1)+7.437(1)+4.075(1)}}{1+e^{-5.418(1)+7.437(1)+4.075(1)}}=0.997$$

$$\mathrm{Pr}ob_{EF,OP}\left(y=1\right)=\frac{e^{-5.418(1)+7.437(1)+1.587(1)}}{1+e^{-5.418(1)+7.437(1)+1.587(1)}}=0.973$$

These probabilities were lower, in particular Pr*ob*_*EF*,*OP*_(*Y* = 1), when the consumer also claimed to discriminate their product selection based on appearance:

$$\mathrm{Pr}ob_{EF,A,B}\left(y=1\right)\frac{e^{-5.418(1)+7.437(1)-2.290(1)+4.075(1)}}{1+e^{-5.418(1)+7.437(1)-2.290(1)+4.075(1)}}=0.978$$

$$\mathrm{Pr}ob_{EF,A,OP}\left(y=1\right)\frac{e^{-5.418(1)+7.437(1)-2.290(1)+1.587(1)}}{1+e^{-5.418(1)+7.437(1)-2.290(1)+41.587(1)}}=0.788$$

For a case in which a consumer appreciated the presence of ecological certifications while simultaneously attributed great importance to product brand and frequently consumed organic products, there was a probability approaching 1, indicating that the said consumer was also strongly concerned about food product safety and health:

$$\mathrm{Pr}ob_{EF,B,OP}\left(y=1\right)\frac{e^{-5.418(1)+7.437(1)+4.075(1)+1.587(1)}}{1+e^{-5.418(1)+7.437(1)+4.075(1)+41.587(1)}}=0.999$$

## 4. Conclusions and Discussion

The calculated values show how an Italian consumer attentive to food safety was equivalent to a Spanish consumer interested in the environmental impact of the products consumed but, unlike the Spanish consumer, the Italian consumer was a frequent consumer of organic products and attributed considerable importance to the brand, in terms of consumption choices. As for the variable relating to product appearance, on the other hand, this had a negative impact on the expected probability of being a consumer attentive to food safety.

In conclusion, it is very likely that an Italian consumer concerned with brands consumes organic products, especially if they appreciate the presence of organic certifications and are also a consumer interested in food safety, and this is in line with other studies conducted in Italy where it is shown how increased awareness of food safety can influence consumer behaviour regarding specific products [37]. It has also been shown that among the several organic food attributes that consumers recognize in organic food, healthiness has been reported as the primary motivation to buy products certified as organic [38].

A Spanish consumer believes that ecological certification on products is important, is very concerned about the environmental impact of the products being consumed and also shows a particular sensitivity to issues pertaining to food safety. As shown by the study of Soler [39], as more accurate information is offered, consumers’ acceptability of labelled organic food products increases.

What characterizes a Spanish consumer who is attentive to food safety is the importance given to the presence of ecological certifications, which, thus, become increasingly important for competitiveness in the food market.

An Italian consumer concerned about food product safety and healthiness is characterized not only by a critical and responsible approach to consumption, attributing importance to ecological certifications and frequent consumption of organic products, but also by the information they try to gather and infer from product brands.

Brands, therefore, play a strong role for Italian consumers. Brand strength seems to offer a global guarantee of the attributes that the Italian consumer is looking for.

This exploratory study has allowed us to highlight sharp differences in the approach to consumption in these two countries, which, despite similar cultures and the same attention to environmental and food safety issues, show marked differences in food products.

Some limitations should be considered on the study conducted. The study was based on a survey that was submitted to a self-selected sample of a moderately small number of Italian and Spanish consumers. The sample can give new intuitions into this dispute but it also stimulates the need for further studies and other investigations.

This research allows us to draw conclusions that could be of interest to other research in light of the findings obtained and could be a potential path to study this topic with a sample stratified by age group and education in order to make inferences to the whole population.

Regulations about brand label should reduce the information asymmetry. Moreover, it is important to increase the awareness of consumers concerning environmental attributes of products and also food product environmental impacts [3].

In particular, what arises from the research is that Italian consumers trust the brand and, therefore, it is very important that claims and messages from branded products are reliable. Italian consumers try to gather and infer food safety attributes from product brands.

Both Spanish consumers and Italian consumers care directly about food safety attributes and environmental certification and consumers in both countries showed increasing attention to environmental certifications. These findings therefore are important, not only for policy makers that can increase the awareness and attention of consumers towards ecological certifications, but also for market operators, because ecological certification has become increasingly important for competitiveness in the food market.

Further research may be conducted in order to investigate these differences in the methods of choice in greater depth. In particular, it may be important to investigate the importance of the brand for Spanish consumers with a larger sample population in order to infer the presence of other food attributes. As for Italian consumers, it may be interesting to understand the confidence in environmental certifications and the possibility that product selection depends mainly on the presence of an environmental or food safety certification, regardless of brand.

## Figures and Tables

**Table 1:** Variables’ description.

Variables	Source	Code
Food Safety	Are you worried about safety food?	No = 0
		Yes = 1
Price	How important is price when you choose and buy food?	Unimportant = 0
		Very important = 1
Aspect	How important is appearance when you choose and buy food?	Unimportant = 0
		Very important = 1
Origin	How important is origin when you choose and buy food?	Unimportant = 0
		Very important = 1
Brand	How important is brand when you choose and buy food?	Unimportant = 0
		Very important = 1
Environmentally Friendly	How important is ecological certification when you choose and buy food?	Unimportant = 0
		Very important = 1
Ecological Certifications	Do you know the meaning of organic certification?	No = 0
		Yes = 1
Organic Products	Do you often purchase organic products?	No = 0
		Yes = 1
Fair Trade	Do you often purchase fair trade products?	No = 0
		Yes = 1
Local Food	Do you often purchase local products?	No = 0
		Yes = 1

**Table 2:** Spanish sample. Output of logistic regression.

	B	S.E.	Forest	df	Sig.	Exp(B)	95% C.I.for EXP(B)	
							Lower	Upper
Price	−0.292	0.390	0.560	1	0.454	0.747	0.348	1.603
Aspect	0.310	0.395	0.618	1	0.432	1.364	0.629	2.957
Origin	0.019	0.409	0.002	1	0.963	1.019	0.457	2.270
Brand	−0.455	0.429	1.125	1	0.289	0.634	0.273	1.471
Environmentally Friendly	1.075	0.399	7.267	1	0.007	2.931	1.341	6.406
Organic Certifications	−0.236	0.503	0.221	1	0.638	0.789	0.295	2.115
Fair Trade	0.299	0.593	0.254	1	0.614	1.348	0.422	4.308
Local Food	0.111	0.408	0.073	1	0.786	1.117	0.502	2.485
Organic Products	0.612	0.505	1.469	1	0.225	1.844	0.686	4.959
Constant	0.105	0.560	0.035	1	0.851	1.111		

**Table 3:** Spanish sample. Output of logistic regression.

	B	S.E.	Forest	df	Sig.	Exp(B)	95% C.I.for EXP(B)	
							Lower	Upper
Environmentally Friendly	1.187	0.364	10.658	1	0.001	3.277	1.607	6.684
Constant	0.480	0.250	3.693	1	0.055	1.615		

**Table 4:** Italian sample. Output of logistic regression by using Forward Stepwise (Likelihood Ratio) Method for selecting variables in the equation.

		B	S.E.	Forest	df	Sig.	Exp(B)	95% C.I.for EXP(B)	
								Lower	Upper
Step 1 ^a^	Environmentally Friendly	3.489	0.482	52,459	1	0.000	32.769	12.746	84.248
	Constant	−1.099	0.385	8147	1	0.004	0.333		
Step 2 ^b^	Environmentally Friendly	5.655	1.073	27,763	1	0.000	285.778	34.870	2342.120
	Brand	3.494	1.054	10,991	1	0.001	32.926	4.172	259.829
	Constant	−4.480	1.119	16,032	1	0.000	0.011		
Step 3 ^c^	Environmentally Friendly	5.203	1.080	23,218	1	0.000	181.849	21.906	1509.596
	Brand	3.579	1.065	11,289	1	0.001	35.842	4.443	289.145
	Organic Products	1.896	0.563	11,349	1	0.001	6.662	2.210	20.080
	Constant	−5.008	1.151	18,941	1	0.000	0.007		
Step 4 ^d^	Environmentally Friendly	7.437	1.555	22,871	1	0.000	1697.377	80.557	35,764.764
	Aspect	−2.290	1.123	4159	1	0.041	0.101	0.011	915
	Brand	4.075	1.084	14,136	1	0.000	58.843	7.033	492.314
	Organic Products	1.587	0.590	7241	1	0.007	4.891	1.539	15.541
	Constant	−5.418	1.163	21,713	1	0.000	0.004		

^a^. Variable(s) entered on step 1: Environmentally Friendly. ^b^. Variable(s) entered on step 2: Brand. ^c^. Variable(s) entered on step 3: Organic Product. ^d^. Variable(s) entered on step 4: Aspect.

## Data Availability

The data are available from the corresponding author.
